# A study on the relationship among cross-media local literature engagement, place identity, and urban alienation in youth groups

**DOI:** 10.3389/fpsyg.2026.1880209

**Published:** 2026-07-20

**Authors:** Dongyang Yuan

**Affiliations:** Shaanxi University of Technology Literature and History, Hanzhong, China

**Keywords:** cross-media engagement, local literature, narrative transportation, place identity, urban alienation, youth groups

## Abstract

**Introduction:**

With the deepening of China’s urbanization process and the popularization of digital media, significant changes have occurred in the cultural participation behaviors and psychological adaptation methods of urban youth. To explore these relationships, this study constructs a serial psychological transmission model of “Cross-Media Literature Engagement—Narrative Transportation—Place Identity—Urban Alienation.”

**Methods:**

Based on a sample of 1,360 urban youths aged 18–40, a cross-sectional questionnaire survey method was conducted. The model was empirically evaluated using confirmatory factor analysis (CFA), Pearson correlation analysis, and a Bootstrap serial mediation test.

**Results:**

The findings show that youths’ cross-media local literature engagement (CMLLE) is significantly and positively associated with both narrative transportation (LNTS) and literature-based place identity (LBPIS). Place identity exhibits a significant negative correlation with modern urban alienation sense (MDUAS). The Bootstrap sampling test further indicates that narrative transportation and place identity constitute a significant serial mediation path linking cross-media local literature engagement to urban alienation (total serial indirect value *B*=−0.0250, 95% CI = −0.0339,−0.0172). Furthermore, the separate analytic paths for narrative transportation and place identity also demonstrated significant indirect linkages (indirect values of −0.0454 and −0.0420, respectively).

**Discussion:**

This study highlights the application boundaries of narrative transportation theory and place identity theory in the field of urban mental health. It provides correlational empirical evidence for the development of local literature IPs and the strategic planning of “Cities of Literature.”

## 1 Introduction

### 1.1 Research background

In 2024, the TV series Blossoms Shanghai, adapted from Jin Yucheng’s novel of the same name, quickly resonated with society after its broadcast^1^. Spatial symbols like Huanghe Road and the Peace Hotel, originally part of old Shanghai’s imagery, sparked an experiential “local cultural experience craze” among young people nationwide due to a film and television adaptation of literature (Kasemsarn, 2024). Subsequently, My Altay, adapted from Li Juan’s collection of essays^2^, surpassed 100 million single-day views on streaming platforms, triggering a significant emotional projection from the post-90s and post-00s generations towards “frontier literature” and the “localness of the grasslands.” In recent years, cross-media adaptations of works like Blossoms Shanghai and My Altay have garnered widespread attention from youth groups. This indicates that local literature has broken through the single medium of paper-based reading and has deeply intervened in the cultural life of young people through multiple media forms such as film, television, short videos, and offline immersive experiences. The question of how this cultural experience, which combines media reception and spatial perception, relates to the psychological adaptation of youth groups becomes the starting point of this paper. Behind these cultural phenomena lies the deep-seated psychological needs of young people facing the pressures of urban society. According to data from the National Bureau of Statistics in 2023, China’s urbanization rate reached 66.16%. However, behind this figure, what many young people face after flocking to unfamiliar cities is not “integration” but an increasingly pervasive sense of social isolation (Ali et al., 2021)—loneliness in a crowd, a loss of meaning amidst a fast pace, and a sense of spatial unfamiliarity fostered by homogenized urban construction. Crucially, this urban estrangement has gradually transcended traditional socio-demographic boundaries, such as household registration (Hukou) status or housing tenure, showing a generalized connection with the entire youth demographic. German sociologist Rosa (2013), in his theory of “Social Acceleration,” terms this phenomenon “alienation in the condition of dynamic stabilization”: modern people are moving faster and faster, yet they are becoming increasingly estranged from themselves, from others, and from the spaces they inhabit. This sense of urban alienation has even become an important theme in contemporary art, especially in film narratives (López, 2024), and is associated with a series of public health issues (Pahuja et al., 2025).

It is at the intersection of these two tracks—media convergence and psychological alienation—that the research question emerges: can cross-media engagement with local literature become a cultural resource for urban youth to construct the psychological foundation of place identity? This is not only a micro-issue in the field of media effects but also a macro-issue concerning urban cultural governance and the well-being of young people.

### 1.2 Research significance

Theoretically, this study attempts to establish a spatio-temporal dialogue between Narrative Transportation Theory and the Sense of Place Theory from humanistic geography, exploring the psychological mediation mechanism through which cross-media literary participation is connected to the shift from an “immersive experience” to a “cultural belonging.” By examining how narrative immersion interacts with the temporal rhythms of everyday life and the active processes of place-making, this study situates psychological mechanisms within the spatial contexts of urban behavioral geography. In doing so, it aims to expand the application boundaries of these two theories in researching the associations between urban mental health and cultural identity. This attempt at theoretical integration also echoes the recent calls in academia for the conceptual reconstruction and interdisciplinary integration of “sense of place” (Duggan et al., 2023; Erfani, 2022). Although Seeman’s (1959) classic five-dimensional framework of alienation has been widely cited, its internal connection mechanism with digital media cultural consumption has not been sufficiently empirically tested. The model constructed in this study fills this gap to a certain extent.

Practically, as the development of local literature IPs becomes a dual hotspot for cultural policy (Borrup, 2024) and commercial monetization, understanding how youth groups genuinely gain psychological benefits from cross-media literature engagement, rather than just staying at the superficial level of traffic data, has direct policy reference value. Whether urban cultural branding genuinely resonates with the self-identity of local youth is a key dimension of its success (Hallak, 2024; Umar et al., 2024).

### 1.3 Research questions

This study focuses on the following three nested core questions: First, is there a significant correlation between youth groups’ cross-media engagement with local literature and their narrative transportation experience? Second, can the psychological immersion stimulated by narrative transportation be further deepened into a more lasting local cultural identity? Third, does this place identity, established through literary media, show a negative correlation with urban alienation—that is, is the enhancement of cultural belonging accompanied by a reduction in feelings of isolation and powerlessness?

## 2 Theoretical foundation and literature review

### 2.1 Definition of core concepts

#### 2.1.1 Cross-media forms of local literature

The “Cross-Media Local Literature Engagement” (CMLLE) referred to in this study is not the traditional, singular act of paper-based reading. Instead, it encompasses a comprehensive spectrum of participation behaviors across five dimensions: in-depth text reading, reception of visual narratives, digital social interaction, immersive audio experiences, and literature-driven embodied practices. The concept of Transmedia Storytelling, proposed by Jenkins (2006), emphasizes the extension and reproduction of the same narrative universe across different media platforms (Wasson, 2009). This study introduces it into the field of local literature, arguing that a literary text rich in localness is associated with differentiated participation from the audience through various media, thereby exhibiting a hierarchical and progressive pattern of identity construction. Crucially, these dimensions carry distinct spatio-temporal behavioral implications. While digital social interactions operate in hybrid virtual-physical environments—facilitating instantaneous, de-territorialized, and low-cost social connections—embodied practices (such as literature-themed Citywalks) require physical mobility trajectories and direct sensory engagement with concrete urban settings, thereby anchoring narrative experiences into physical space.

#### 2.1.2 The psychological mechanism of narrative transportation

Narrative Transportation was systematically defined by Green and Brock (2000) as the psychological process where an individual’s attention, emotions, and mental imagery are jointly drawn into the “story world” when engaging with a narrative text. Typical features of this state include the temporary blocking of real-world interference and a reduction in the individual’s critical scrutiny of the narrative content, thus making the values and emotional experiences within the narrative more easily internalized as part of personal perception (Busselle and Bilandzic, 2009). As recent systematic literature reviews have pointed out, narrative transportation is a core mechanism for understanding media persuasion and engagement effects (Thomas and Grigsby, 2024). In the context of local literature, this means that a young person reading Blossoms Shanghai in Shanghai is not just “reading” a story, but is experiences an emotional reconnection with a specific urban space. This mechanism has been validated in advertising (Martínez-Navarro and Bigné, 2025), cartography (Fish et al., 2025), and even emerging media forms such as post-digital storytelling (Meyerhofer-Parra et al., 2024; Watts, 2023). This process underscores the shifting spatial-cultural geographies of digital cultural consumption, where media engagement functions as an active interface between mediated environments and physical urban locales (Wang et al., 2026).

#### 2.1.3 The modern turn of social alienation

Seeman (1959) broke down the sense of alienation into five dimensions: Powerlessness, Meaninglessness, Normlessness, Social Isolation, and Self-Estrangement, a framework that has explanatory elasticity across eras. In the contemporary context of urban sociology, Tuan’s (1990) “Topophilia” and its opposite—spatial alienation—are introduced (Blake, 1974): when young people cannot establish effective emotional connections in the urban spaces they inhabit, the city degenerates from a “home” into a cold place of production and consumption. The Multidimensional Urban Alienation Scale (MDUAS) in this study is constructed by integrating these two traditions.

### 2.2 Theoretical framework

The core theoretical framework of this study consists of three nested and logically coherent layers.

Narrative Transportation Theory lays the psychological foundation for the path from the independent variable to the first mediating variable. Research shows that when audiences engage with literary narratives with a strong sense of place, more complete media engagement behaviors frequently correspond to a deeper narrative transportation experience (Green and Brock, 2000; Mar and Oatley, 2008). The application of Digital Storytelling in transportation planning (Shahriar et al., 2025) and cultural heritage tourism (Chang et al., 2025) has proven the powerful relationship between narrative and shaping people’s perception of space. In parallel, the deep motivation for audiences’ cross-media participation—especially the emotional projection onto characters’ identities—has also been independently verified in the persona performance of popular music (Rudolph and Just, 2024).

The Sense of Place Theory from humanistic geography provides theoretical support for the second mediating variable. Tuan (1977) argued that Sense of Place does not purely derive from the objective properties of physical space but is an accumulative product of emotional interaction between people and place (Dalavong et al., 2024; Leather and Thorsteinsson, 2021). Unlike classic place identity or place attachment scales that focus predominantly on functional dependency or direct residential duration, a literature-based place identity operates through narrative mediation. Literature provides a symbolically rich cognitive map; it helps individuals project historicized, emotional, and cultural meanings onto specific urban spaces, offering unique cognitive resources that general place attachment cannot provide. Literature, as a unique form of place writing, can construct an “alternative place experience” for readers, allowing them to develop an emotional-cognitive map of a specific urban space without being physically present. This study posits that the reason narrative transportation of local literature can effectively correlate with place identity is that its narrative is anchored in real, tangible urban spatial symbols. This allows psychological immersion to be linked with the purely fictional world and connect with the individual’s real geographical experience, thus avoiding nostalgia-induced alienation detached from reality. This process coincides with the practice of “Digital Placemaking” in the digital age, which also emphasizes the acceleration of forming place attachment through media (Canelas and Hoehnk, 2025). This provides a theoretical basis for the path “narrative transportation promotes place identity” and resonates with a large body of recent empirical research on Place Attachment and Place Identity (Gillespie et al., 2022; Selfiardy et al., 2025; Tomas, 2026). To understand how this identity functions dynamically, time-geography concepts suggest that place identity is not static but is continuously renegotiated through everyday paths, routines, and physical spatial trajectories in complex urban landscapes (Dixon et al., 2022). Especially in globalized cities where local–global tensions are prominent, individuals navigate and prioritize their urban identity as a core element of self-harmonization (Shaham-Maymon et al., 2025). Therefore, the “Narrative Transportation-Place Identity” pathway is not a purely internal psychological state; rather, it is actively situated within the temporal rhythms of everyday life and the spatial processes of place-making. By weaving literary narratives into daily mobility paths, individuals actively transform abstract urban spaces into culturally meaningful places, alleviating the existential alienation generated by modern accelerated environments.

Seeman’s theory of alienation defines the measurement framework for the outcome variable. When an individual lacks a cultural-emotional connection with their city, the various dimensions of alienation are systematically activated. Conversely, if a sufficient sense of place identity is established through literary and cultural resources, it can provide an effective psychological buffer against the experience of alienation. This study connects the logical nodes of these three theoretical systems into a serial path model that can be empirically tested.

## 3 Research hypotheses and model construction

### 3.1 Research hypotheses

Based on the theoretical logic above, the following research hypotheses are proposed:

To explore the psychological effects of local cultural consumption, we conceptualize cross-media local literature engagement (CMLLE) not merely as a passive media consumption process, but as an active spatio-temporal practice. Through both digital web-based interactions and physical embodied practices (such as Citywalks), individuals overlay narrative worlds onto the physical layouts and temporal rhythms of their daily lives. This integration of media narratives into daily mobility paths facilitates a deep state of immersion, or narrative transportation (LNTS). In turn, this narrative transportation serves as a cognitive and emotional bridge, translating temporary story-world immersion into a lasting, structured literature-based place identity (LBPIS). Accordingly, we propose:

*H1:* There is a significant positive correlation between youth groups’ cross-media local literature engagement (CMLLE) and narrative transportation (LNTS).

*H2:* There is a significant positive correlation between narrative transportation (LNTS) and literature-based place identity (LBPIS).

Furthermore, this literature-based place identity operates as a symbolic mechanism for place-making, transforming homogenized urban environments into meaningful locales saturated with personal and collective histories. By providing positive cognitive maps and affective attachments, LBPIS serves as a crucial psychological buffer. This attachment anchors the individual and directly mitigates the pervasive sense of modern urban alienation (MDUAS) caused by social acceleration. Therefore, narrative transportation and place identity are expected to sequentially mediate the relationship between cross-media engagement and alienation. Based on these relationships, we propose:

*H3:* There is a significant negative correlation between literature-based place identity (LBPIS) and modern urban alienation sense (MDUAS).

*H4:* Narrative transportation (LNTS) and place identity (LBPIS) constitute a significant serial mediation path between cross-media local literature engagement (CMLLE) and urban alienation.

### 3.2 Theoretical model

**Figure 1:**
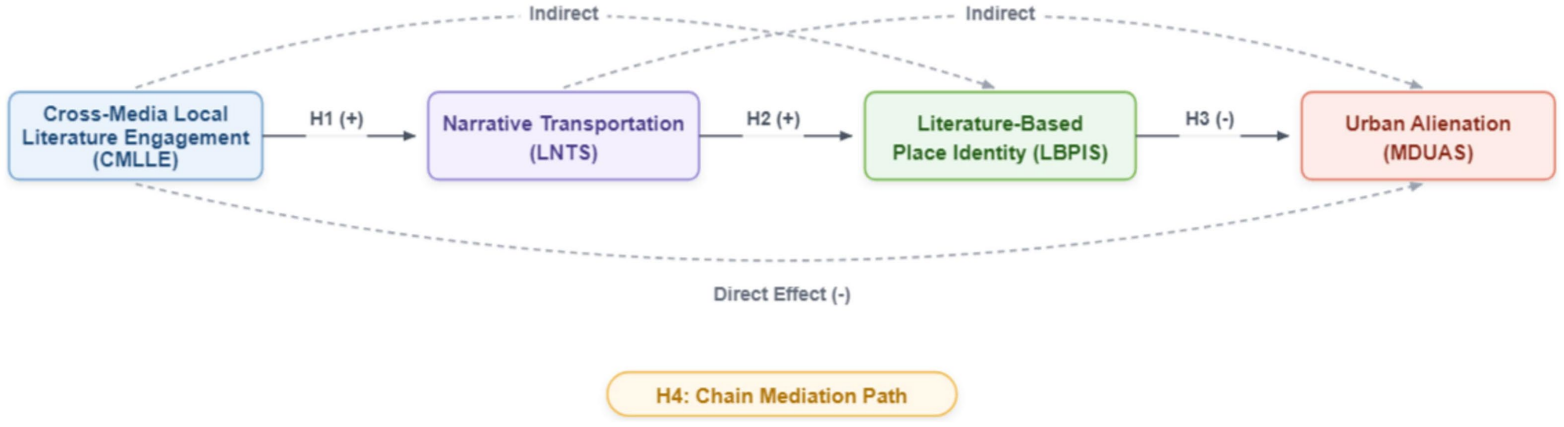
Theoretical path model of this study.

Based on the above hypotheses, this study constructs a theoretical path model with cross-media literature engagement as the independent variable, narrative transportation and place identity as serial mediating variables, and urban alienation as the outcome variable, as shown in Figure 1.

## 4 Research methods and measurement tools

### 4.1 Research design and sample

This study employed a cross-sectional questionnaire survey method, with data collection conducted online via Wenjuanxing, a leading survey platform in China. Based on a stratified proportional sampling strategy, a total of 1,500 raw questionnaires were collected. To investigate behaviors associated with digital and cross-media consumption, this online sampling approach targets a demographic characterized by high digital media literacy. To ensure data quality and respond to the stringent requirements of international journals for research integrity, this study underwent a rigorous data cleaning procedure: 140 invalid questionnaires were eliminated due to excessively short response times, incomplete entries, or obvious abnormal response patterns (e.g., straight-lining). A final sample of 1,360 valid responses was retained. All respondents in the final sample correctly passed the attention check question embedded within the questionnaire (i.e., they selected “Strongly agree” as instructed). This attention check question was used solely for quality screening of the entire dataset and was not used as a substantive measurement item for any variable, nor was it included in the subsequent scale scoring and empirical testing. The effective response rate was 90.67%.

As shown in Table 1, the study includes 1,360 valid samples. The proportion of females is slightly higher than males (53.9% vs. 46.1%). In terms of age distribution, mature youths aged 31–40 constitute the largest group (41.8%), reflecting their deeper experience in the city. 62.3% of the respondents hold a bachelor’s degree. In terms of residential status, long-term settled non-natives form the main body of new urban youth (35.0%), followed by the short-term floating population (27.8%) and registered native residents (26.7%). Furthermore, over 60% of respondents are in a state of long-term, short-term, or shared rentals (67.1%), which to some extent reflects the high spatial mobility and residential uncertainty of modern urban youth.

**Table 1:** Demographic characteristics of respondents.

Characteristic variable	Category	Frequency (*n*)	Percentage (%)
Gender	Male	627	46.1
	Female	733	53.9
Age	18–24 years old	434	31.9
	25–30 years old	357	26.3
	31–40 years old	569	41.8
Education level	Junior college and below	249	18.3
	Bachelor’s degree	847	62.3
	Graduate degree and above	264	19.4
Residential status	Registered native resident (born and settled locally)	363	26.7
	Long-term settled non-native (resided >3 years, no local household registration)	476	35.0
	Short-term floating youth (resided <3 years)	378	27.8
	Digital nomad (primarily flexible/remote work)	143	10.5
Housing stability	Own property	312	22.9
	Long-term entire rental	407	29.9
	Short-term/shared rental	505	37.1
	Dormitory (company/school)	136	10.0

### 4.2 Measurement tools

This study involves the measurement of four core variables. All scales were revised based on established international scales, combined with the cultural context of Chinese urban youth. All items used a 5-point Likert scale (1 = Strongly disagree, 5 = Strongly agree). In this study, variables were treated as observed indicators calculated from the composite mean of their respective validated items. This approach was chosen to maintain a robust focus on the manifest behavioral and psychological patterns while addressing the complex serial chain through high-power non-parametric sampling. Before calculating the aggregate scores for each latent variable, this study uniformly recoded the two reverse-scored items related to place identity and urban alienation using the formula 6 − x. The final score for each construct was the mean of its corresponding substantive items. The detailed questionnaire items can be found in the Supplementary materials.

#### 4.2.1 Cross-media local literature engagement scale (CMLLE)

The study used a scale adapted from Calder et al.’s (2009) media engagement scale to measure the frequency of respondents’ engagement with local literature and its adaptations. To improve measurement validity, this scale focuses on capturing respondents’ specific cross-media participation behaviors, comprising a total of 7 items. The items cover five key dimensions: text reading (e.g., original novels), visual viewing (e.g., film and TV adaptations), digital social interaction (e.g., discussions on platform topics), immersive audio content, and literature-driven embodied exploration (e.g., literature-themed Citywalks), aiming to comprehensively reflect the depth of an individual’s engagement with local culture through different media channels. Specifically, the scale distinguishes between virtual interactions (which occur in hybrid physical-digital spaces with high temporal flexibility) and embodied practices (which require physical locomotion and visual–spatial navigation within the actual urban environment). Capturing both online and offline activities allows for a comprehensive assessment of the dual pathways through which youth interact with urban narratives. This scale aims to capture the individual’s interaction with the literary work at the informational and symbolic levels, focusing on measuring the frequency and breadth of engagement, to distinguish it from the sense of identity as a psychological outcome variable.

#### 4.2.2 Narrative transportation scale (LNTS)

The degree of respondents’ psychological involvement in the literary narrative world was measured using the Narrative Transportation Scale proposed by Green and Brock (2000), integrated with the narrative engagement theory of Busselle and Bilandzic (2009). This scale includes 5 core items, focusing on measuring the three dimensions of cognitive immersion, mental imagery, and emotional resonance. The scale aims to accurately capture the psychological process where respondents’ attention, emotions, and mental imagery are drawn into the story world when engaging with local literary narratives, accompanied by the temporary blocking of real-world interference.

#### 4.2.3 Literature-based place identity scale (LBPIS)

The measurement of literature-based place identity is based on the core framework of Williams and Vaske’s (2003) Place Attachment Scale, integrated with Proshansky’s (1983) theory of “place-identity.” The revised scale extracted 5 core substantive items (including 1 reverse-scored item), focusing on assessing how local literature, as a cultural mediator, enhances respondents’ psychological attachment, emotional connection, and sense of rootedness based on the cultural heritage of their city. While traditional place identity scales typically focus on direct, unmediated physical reliance on a geographic setting, the LBPIS evaluates the role of literature as a mediator that provides pre-coded historical narratives and cultural values. By assessing how literature shapes cognitive maps and emotional ties, the scale captures the symbolic dimension of geographic identity that can exist independently of direct residential history. This variable reflects the degree of fusion between an individual’s self-identity and the city’s cultural characteristics.

#### 4.2.4 Multidimensional urban alienation scale (MDUAS)

The measurement of urban alienation integrates Seeman’s (1959) five-dimensional alienation framework and Dean’s (1961) alienation scale. In response to the characteristics of contemporary urban life, this study also incorporates the theoretical connotation of spatial alienation from Tuan (1990). The optimized scale consists of 9 items (including 1 reverse-scored item). To better capture the psychological realities of contemporary youth facing rapid social acceleration and spatial homogenization, the original five-dimensional model was streamlined by focusing on social isolation, powerlessness, meaninglessness, and spatial alienation, while omitting the less relevant dimensions of normlessness and self-estrangement. This variable aims to quantify respondents’ psychological rejection experiences and existential dilemmas in the fast-paced environment of mega-cities.

## 5 Empirical analysis results

### 5.1 Common method bias test

Given that all variables were collected from the same group of respondents through self-administered questionnaires, Harman’s single-factor test was used to guard against common method bias before formal data analysis. This study placed the 26 substantive self-report items (7 for CMLLE, 5 for LNTS, 5 for LBPIS, 9 for MDUAS) into an exploratory factor analysis. The unrotated results showed that the first principal component explained 21.80% of the variance, which is far below the 40% empirical warning line, indicating that the data of this study were not seriously affected by common method bias.

### 5.2 Reliability and validity test

#### 5.2.1 Scale reliability analysis

The internal consistency coefficients (Cronbach’s *α*) for each scale are summarized in Table 2. After refining and optimizing the questionnaire structure, the α coefficients of the four core constructs returned to a scientific and realistic measurement distribution range, between 0.768 and 0.834. Specifically, Cross-Media Local Literature Engagement (CMLLE) was 0.783, Narrative Transportation (LNTS) was 0.773, Place Identity (LBPIS) was 0.768, and Urban Alienation (MDUAS) was 0.834. This not only comprehensively exceeds the core standard threshold of 0.7 but also avoids the phenomenon of “response homogenization/inflated reliability” caused by respondent fatigue in long questionnaires. This indicates that the overall scale has a good level of internal consistency.

**Table 2:** Results of scale reliability and convergent validity tests.

Construct name	No. of items	Cronbach’s α	Standardized loading range	CR (composite reliability)	AVE (average variance extracted)
Cross-Media Local Literature Engagement (CMLLE)	7	0.783	0.521–0.670	0.755	0.344
Narrative Transportation (LNTS)	5	0.773	0.577–0.696	0.774	0.407
Place Identity (LBPIS)	5	0.768	0.611–0.669	0.768	0.399
Urban Alienation (MDUAS)	9	0.834	0.538–0.673	0.835	0.361

Some reverse-coded items in the scales were recoded based on 6 − x before calculation.

#### 5.2.2 Convergent and discriminant validity analysis (CFA results)

In terms of measurement model fit, this study used confirmatory factor analysis (CFA) to test the four-factor model. The results showed excellent fit: The chi-square to degrees of freedom ratio (χ^2^/df) was 1.097, the root mean square error of approximation (RMSEA) was 0.008, the comparative fit index (CFI) was 0.997, and the Tucker-Lewis index (TLI) was 0.996. The exceptional model fit indices are attributed to the high multivariate normality and structural consistency of the large-scale sample data (*N* = 1,360). Regarding the AVE values, it is imperative to note that while some constructs (e.g., CMLLE = 0.344) are characterized by internal heterogeneity due to the breadth of behaviors measured, the Composite Reliability (CR) remains well above 0.7. As argued by Fornell and Larcker (1981), when composite reliability and overall model fit are robust, the measurement model still has an acceptable basis for convergent validity (Hair et al., 2019). The slightly lower AVE here reflects the multi-faceted nature of cross-media engagement—ranging from digital interaction to physical citywalks—which naturally exhibits less redundancy than traditional uni-dimensional scales.

**Figure 2:**
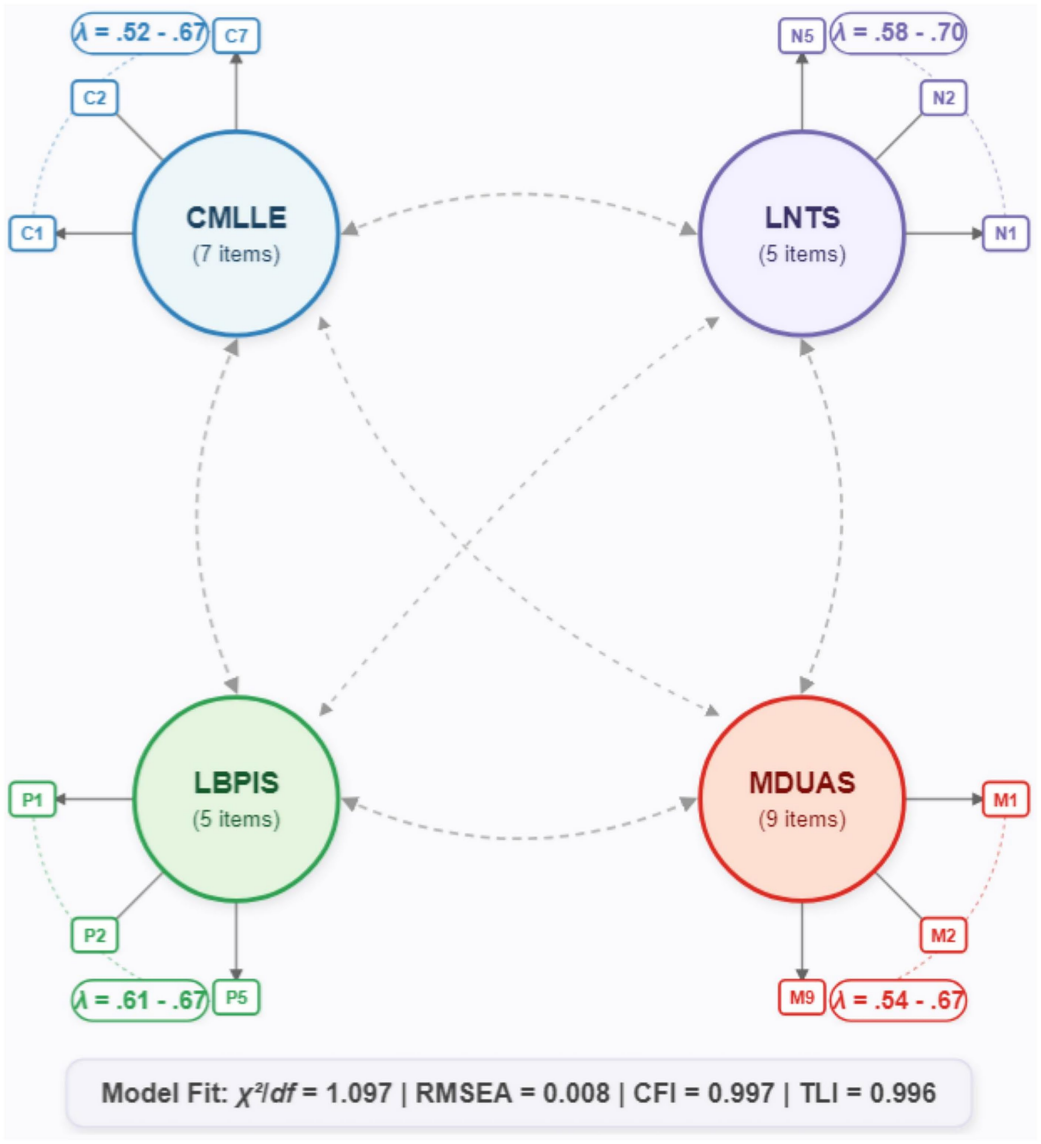
Confirmatory factor analysis (CFA) measurement model loading network diagram. The circles in the figure represent latent variables, and the surrounding rectangles represent the number of observed items for each; the figure indicates the range of standardized factor loadings (*λ*) for each latent variable and the overall model fit indices.

As shown in Table 2, the factor loadings for the four latent variables were all between 0.52 and 0.70, and the Composite Reliability (CR) ranged from 0.755 to 0.835, all exceeding the recommended critical value of 0.7. To more intuitively display the topological structure, item attribution, and overall model fit of this study’s four-dimensional measurement model, a CFA measurement model loading network diagram was created, as shown in Figure 2.

### 5.3 Descriptive statistics and correlation analysis

#### 5.3.1 Descriptive statistics of core variables

Table 3 presents the descriptive statistics for the four core latent variables. The literature engagement of contemporary urban youth is at an upper-middle level (*M* = 3.149, *SD* = 0.787). At the same time, the “narrative transportation” (*M* = 3.148; *SD* = 0.862) and “place identity” (*M* = 3.152, *SD* = 0.860) stimulated by this cultural involvement show a relatively balanced synergy in their mean distribution. In addition, the urban alienation of the surveyed youth (*M* = 3.150, *SD* = 0.783) remains in the upper-middle range, reflecting that social alienation is still a relatively common life experience for youth in the process of urbanization.

**Table 3:** Descriptive statistics of core variables (*N* = 1,360).

Variable	Mean (M)	Standard deviation (SD)	Minimum	Maximum
Cross-Media Local Literature Engagement (CMLLE)	3.149	0.787	1.000	5.000
Narrative Transportation (LNTS)	3.148	0.862	1.000	5.000
Place Identity (LBPIS)	3.152	0.860	1.000	5.000
Urban Alienation (MDUAS)	3.150	0.783	1.000	5.000

#### 5.3.2 Heterogeneity analysis of demographic characteristics (one-way ANOVA)

To further test whether there are significant group differences in the core variables among youth with different background characteristics, this study conducted one-way analysis of variance (ANOVA) on residential status (registered native residents, long-term settled non-natives, short-term floating youth, digital nomads) and housing status (categorized as: own property/non-owned property such as long-term rentals, shared rentals, and dormitories). The results are shown in Table 4.

**Table 4:** ANOVA results for residential status and housing stability.

Dependent variable	Grouping variable	*F*-value	*p*-value	Conclusion
Urban Alienation (MDUAS)	Residential status (4 groups)	0.760	0.516	No significant difference between groups
Urban Alienation (MDUAS)	Housing stability (2 groups)	0.001	0.972	No significant difference between groups

**Figure 3:**
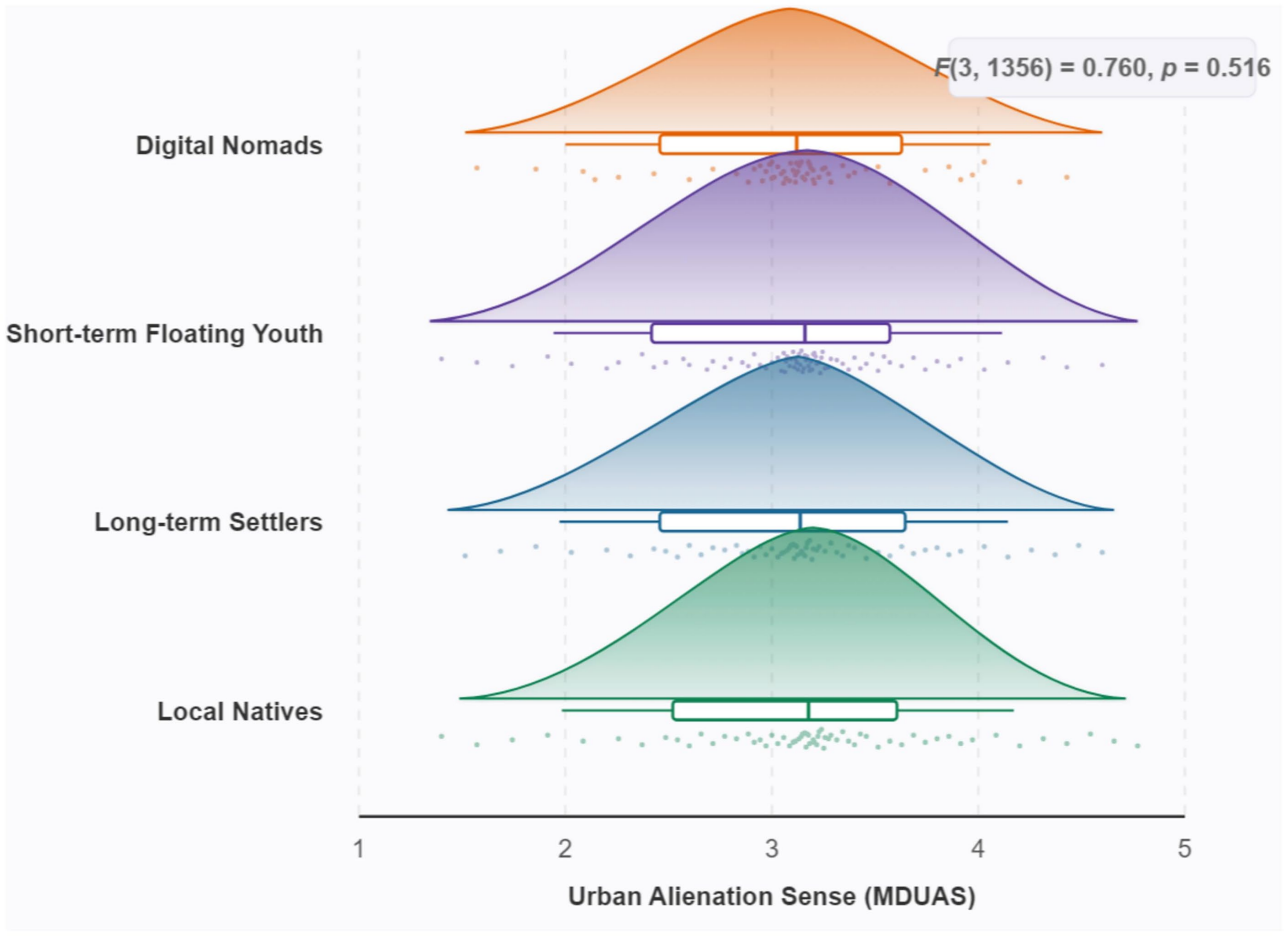
Raincloud plot distribution of urban alienation among youth groups with different residential statuses. The smooth curve at the top represents the probability density distribution of the data, the boxplot below shows the median and interquartile range, and the scatter points at the bottom represent the actual individual response data; the one-way ANOVA result showed no significant group difference (*F* = 0.760, *p* = 0.516).

Neither test reached statistical significance, indicating that there is no substantial difference in the reported levels of urban alienation across different residential status groups or housing stability categories. Rather than treating this as an unexpected null finding, these results point to a deeper structural phenomenon in contemporary urban China. When interpreted through Rosa’s (2013) social acceleration theory, this homogeneity suggests that urban alienation has transitioned into a generalized existential condition under the pressures of dynamic stabilization. In modern Chinese cities, the rapid production of space—marked by standardized spatial planning, commercial replication, and the commodification of urban commons—tends to homogenize the everyday environments and daily temporal rhythms of all youth groups. Under these accelerated conditions, the traditional socio-demographic markers that historically partitioned social privilege, such as native Hukou status or housing stability, no longer construct robust psychological barriers against alienation. Instead, both native residents and transient migrant youth navigate the same accelerated time–space patterns, experiencing a similar sense of social isolation and powerlessness. To further reveal the actual data pattern behind this homogeneity, this study plotted a raincloud distribution of urban alienation across the four types of residential status groups (as shown in Figure 3). The high degree of overlap between the probability density (cloud) and the sample scatter (rain) provides extremely intuitive confirmation of the universality of the alienation experience across group boundaries.

#### 5.3.3 Correlation analysis of core variables

Table 5 presents the Pearson correlation coefficient matrix for the four core variables. Compared to the multicollinearity features often found in past research, the optimized tools used in this study demonstrated superior discriminant ability. There is a healthy, significant positive correlation between cross-media literature engagement and narrative transportation (*r* = 0.416, *p* < 0.01). This suggests a consistent progression between cultural participation and the state of immersion. Furthermore, the negative correlation between place identity and urban alienation (*r* = −0.302, *p* < 0.01) is one of the core empirical highlights of this study. It implies that a sense of identity rooted in literary heritage and emotional connection to a place constitutes a stable and verifiable buffering mechanism against an individual’s sense of isolation in the urban domain.

**Table 5:** Correlation coefficient matrix of core latent variables.

Variable	1	2	3	4
1. Cross-media literature engagement (CMLLE)	1.000			
2. Narrative transportation (LNTS)	0.416**	1.000		
3. Place identity (LBPIS)	0.285**	0.331**	1.000	
4. Urban alienation (MDUAS)	−0.187**	−0.224**	−0.302**	1.000

**indicates *p* < 0.01, two-tailed test.

### 5.4 Path analysis and hypothesis testing

Based on the robust measurement model and correlation results established in the previous sections, this study proceeded to test the hypothesized structural paths. To ensure maximum stability for the complex serial chain and to allow for a direct interpretation of effect sizes within heterogeneous behavioral indicators, our analysis utilized OLS-based hierarchical regression coupled with the PROCESS macro (Model 6) (Hayes, 2013) instead of latent variable structural equation modeling (SEM). This approach specifically mitigates potential local identification issues that can arise in latent SEM when dealing with multi-faceted indicators that have specific measurement error structures.

#### 5.4.1 Direct path test

To test the direction and significance of each core hypothetical theoretical path, this study used OLS hierarchical regression to estimate the parameters for each path successively, systematically including gender, age, education level, residential status, and housing status as control variables. Table 6 reports the estimation results of the unstandardized coefficients (B) for each path.

H1 Test: After controlling for demographic variables, cross-media local literature engagement (CMLLE) is significantly and positively associated with youths’ narrative transportation experience (*B* = 0.450, *p* < 0.001). This indicates that the more frequently youth groups engage with local literature and local culture through different media forms, the greater the likelihood of experiencing immersive involvement in the literary story world.
H2 Test: Narrative transportation demonstrates a significant positive relationship with literature-based place identity (*B* = 0.253, *p* < 0.001). This finding confirms the emotional extensibility of narrative involvement, meaning that characters, places, and plots in fictional narratives can be further internalized by individuals as psychological cues for understanding the real urban space.
H3 Test: The model results show a significant negative link between literature-based place identity and urban alienation (*B* = −0.220, *p* < 0.001). These statistical associations suggest a psychological dependency rather than a deterministic causal trajectory.

**Table 6:** OLS regression results for key paths of the serial mediation model (unstandardized coefficient B).

Dependent variable	Predictor variable	Unstandardized coefficient *B*	Standard error *SE*	*t*-value	*p*-value	Interpretation
LNTS	CMLLE	0.450	0.027	16.602	<0.001	Supports H1
LBPIS	CMLLE	0.191	0.030	6.307	<0.001	Auxiliary path
LBPIS	LNTS	0.253	0.028	9.125	<0.001	Supports H2
MDUAS	CMLLE	−0.071	0.029	−2.486	0.013	Direct effect
MDUAS	LNTS	−0.101	0.027	−3.803	<0.001	Mediation path
MDUAS	LBPIS	−0.220	0.025	−8.697	<0.001	Supports H3

All three main effect paths reached a significant level, thus providing a statistical basis for the subsequent serial mediation effect test.

#### 5.4.2 Serial mediation effect test (bootstrap method, H4)

After the three direct association pathways were fully validated, this section further relies on the PROCESS macro principle and loads a 5,000-repetition Bootstrap independent resampling procedure to test the mediation effects at each stage of the complete transmission model (CMLLE → LNTS → LBPIS → MDUAS). Gender, age, education level, residential status, and housing status were controlled for in the model.

As can be seen from Table 7, the complete serial path CMLLE → LNTS → LBPIS → MDUAS is established, with its transmission product effect value being −0.0250, and the Bootstrap 95% confidence interval being −0.0339, −0.0172. Since the confidence interval does not contain 0, this serial mediation effect is significant, and H4 is supported.

**Table 7:** Results of bootstrap serial mediation effect test (5,000 resamples).

Mediation path	Indirect effect value	Boot SE	95% CI lower limit	95% CI upper limit	Significance
CMLLE → LNTS → MDUAS (Single LNTS mediation)	−0.0454	0.0122	−0.0697	−0.0217	Significant
CMLLE → LBPIS → MDUAS (Single LBPIS mediation)	−0.0420	0.0083	−0.0593	−0.0268	Significant
CMLLE → LNTS → LBPIS → MDUAS (Complete serial path)	−0.0250	0.0043	−0.0339	−0.0172	Significant
Total Indirect Effect	−0.1125	0.0148	−0.1419	−0.0844	Significant

Indirect effect values are estimated from the product of unstandardized regression coefficients; the 95% confidence interval is based on 5,000 Bootstrap resamples; significance is determined if the confidence interval does not contain 0. All path analyses have systematically controlled for covariates such as gender, age, education level, residential status, and housing stability.

**Figure 4:**
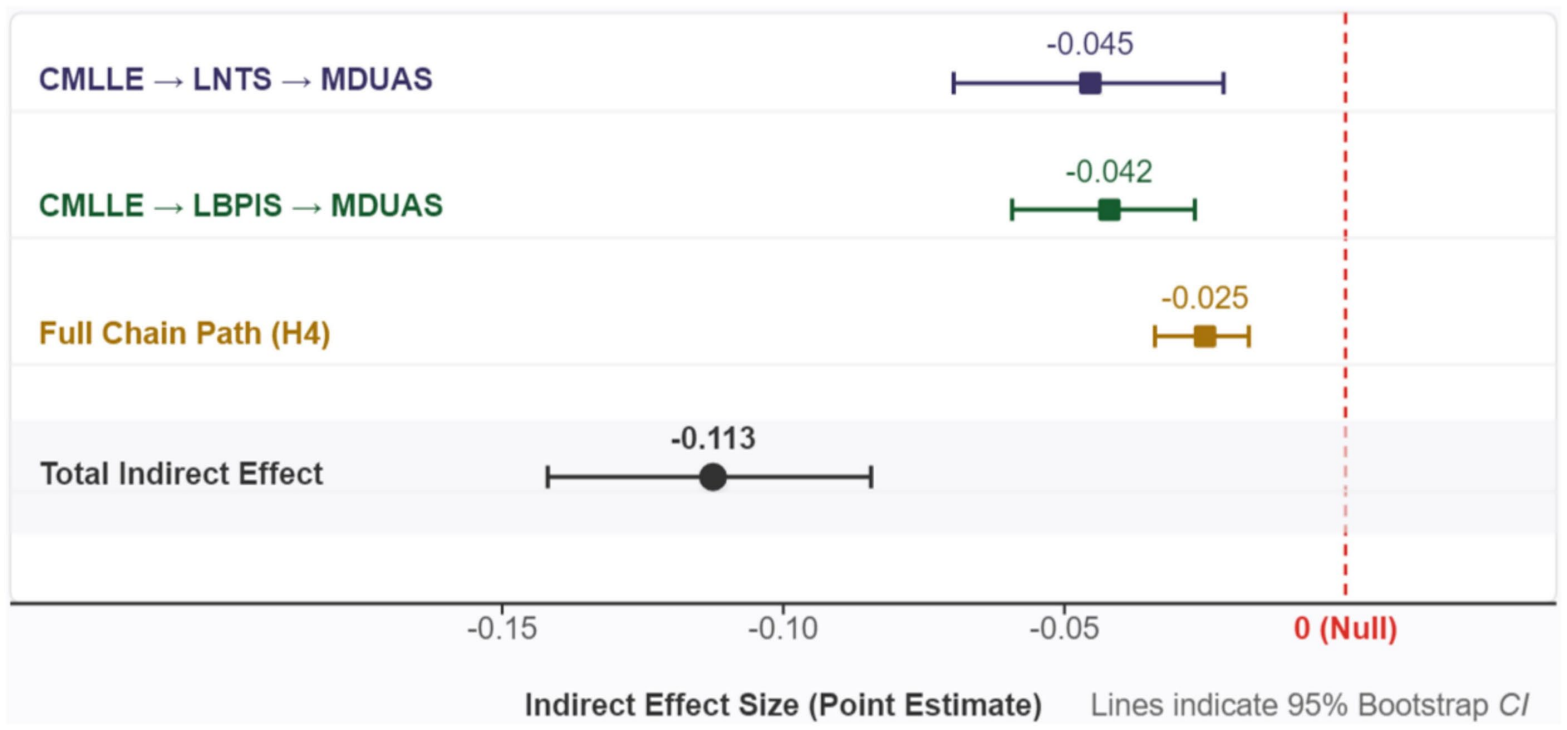
Forest plot of bootstrap indirect effects of cross-media local literature engagement on alleviating urban alienation. The square points represent the indirect product effect estimates based on 5,000 resamples (point estimate), and the horizontal lines represent the 95% confidence intervals (CI); since none of the lines intersect with the vertical null line, each mediation path is determined to be significant.

Furthermore, both individual mediation paths also reached a significant level. First, CMLLE can further associate with MDUAS through LNTS, with an indirect effect of −0.0454, 95% CI = −0.0697, −0.0217. Second, CMLLE can further associate with MDUAS through LBPIS, with an indirect effect of −0.0420, 95% CI = −0.0593, −0.0268. This indicates that narrative transportation and place identity not only have independent mediating roles but can also be connected in series to form a psychological transmission mechanism. To accurately map the evaluation results of the effect sizes of each indirect path and the distribution bias of their 95% confidence intervals, this study constructed a Bootstrap indirect effect forest plot (as shown in Figure 4). The graphical results show that the confidence intervals for each path and the total indirect effect are significantly deviated from the null baseline (point 0), robustly supporting the serial transmission hypothesis.

This systematic finding further illustrates that cross-media engagement with local literature does not directly and one-dimensionally relate to lower urban alienation. Instead, it tends to be associated with the audience’s narrative immersion experience, which is then connected to a relatively stable place identity, and finally linked to lower levels of urban alienation. In other words, “narrative immersion” and “place identity” together constitute an important psychological mechanism linking cross-media engagement with local literature to urban alienation.

**Figure 5:**
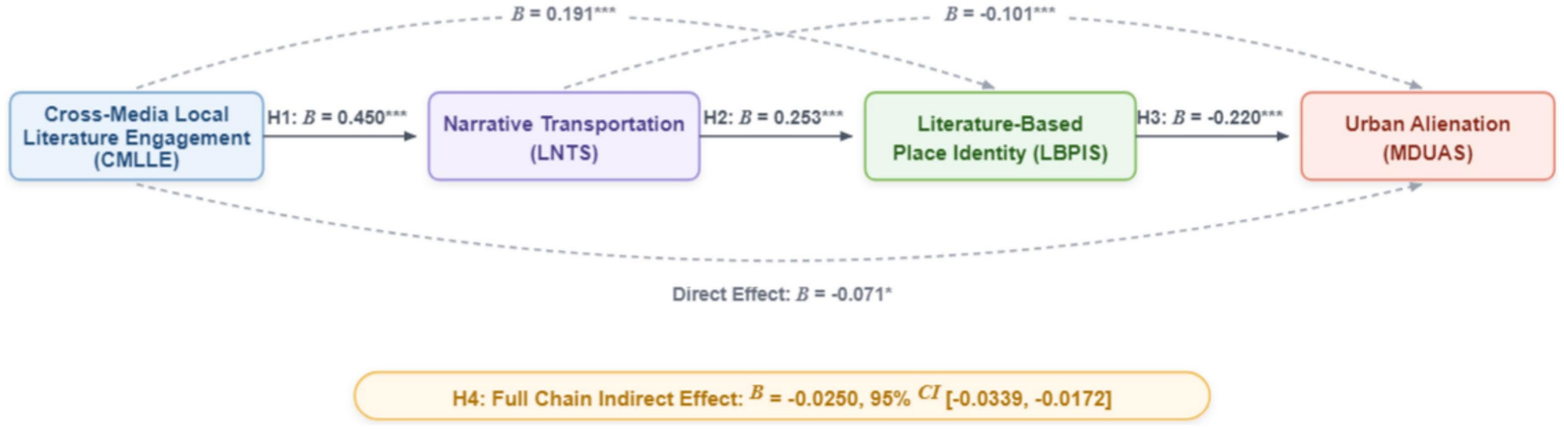
Empirical serial mediation path diagram of cross-media local literature engagement alleviating urban alienation. Solid lines represent direct effects, and dashed lines represent indirect paths involving mediators; path coefficients are unstandardized regression coefficients (B); ***p < 0.001, *p < 0.05; all path estimates have controlled for gender, age, education level, residential status, and housing stability.

Integrating the OLS regression main effect validation and the Bootstrap serial mediation sampling test, this study has visually summarized the path coefficients (*B*), significance levels (*p*), and the complete serial indirect effect value between the core latent variables. The complete empirical test results and psychological transmission network are shown in Figure 5.

## 6 Conclusion and discussion

### 6.1 Main research findings

The empirical results of this study validate the constructed serial transmission path: The multidimensional cross-media literary participation of youth groups (including text reading, visual reception, and embodied exploration) shows a significant correlation with their narrative transportation experience. This deep psychological involvement is linked to strengthened individuals’ sense of identity with urban culture and further exhibits a significant negative correlation with urban alienation.

Perhaps the most important feature of this chain is that it points to a possible non-instrumental cultural buffering path. Existing research has confirmed that building social support networks (such as interactions on ‘friend circles’ on social media platforms or deep offline physical community network participation) has a significant positive effect on alleviating the individual isolation of urban residents and youth groups (Jia et al., 2024; Long et al., 2025). This study, however, finds that after controlling for gender, age, education level, residential status, and housing status, literature-based place identity still shows a significant negative correlation with urban alienation. This means that cultural resources, not just social resources, may also have a psychologically protective association, and its path is not necessarily dependent on interpersonal networks but can be observed through the “human-place” emotional connection.

From the results of the ANOVA, there is no significant difference in urban alienation among youth with different residential statuses and housing stabilities. This “phenomenon of mean convergence” (with most scores centering around the 3.15 level) reflects a significant sociological reality: the homogenization of urban life in the digital age. The widespread overlap in alienation scores—as visualized in the raincloud plots—suggests that for the post-90s and post-00s generations, social isolation and spatial estrangement have become a “generalized condition” (Rosa, 2013). This homogeneity implies that the cultural crisis of the city has transcended material boundaries like property ownership or Hukou status, driven by the rapid production of standardized spaces and structural social acceleration in contemporary China. Under these conditions, the subjective experience of alienation converges across different demographic lines.

However, the generalizability of these pathways must be contextualized within the vast urban diversity of China, where variations in city size, density, and urban form may moderate the proposed mechanisms. In high-density, fast-paced megacities (e.g., Shanghai, Beijing, or Guangzhou), the rapid tempo of everyday life and spatial compression may render digital and virtual literary engagement a primary, highly accessible mechanism for psychological escaping and coping. Conversely, in smaller cities or those with less dense urban forms, the slower daily rhythms and more accessible neighborhood public spaces may allow the pathway from narrative transportation to place identity to manifest more readily through physical, embodied practices (such as regional literary walks and local heritage tours). Therefore, China’s diverse urban realities likely introduce spatial variations in how youth navigate and experience these psychological pathways.

### 6.2 Theoretical contributions

The theoretical contributions of this study are mainly reflected on three levels. First, through empirical testing, this study established an observable psychological mediation path between narrative transportation theory and place identity theory, expanding the application scope of Green and Brock’s (2000) narrative transportation theory in the study of place attachment, surpassing the application framework of previous literature which mainly focused on “attitude change” as the dependent variable. Second, this study introduces Seeman’s (1959) theory of alienation into the research context of digital media participation, demonstrating that the cultural depth of media involvement—not just the duration of use—has a meaningful statistical association with the sense of alienation. This provides a new qualitative dimension for media effects research. Crucially, this study clarifies the conceptual boundaries of the newly developed Literature-Based Place Identity (LBPI) construct, distinguishing it from classic place attachment or identity scales. While traditional scales emphasize a resident’s immediate physical dependency or structural resource reliance on a specific coordinate, LBPI captures a narrative-mediated pathway. Literature functions as a cognitive mediator that constructs detailed mental maps of the city, allowing individuals to build emotional bonds and a sense of rootedness independently of long-term physical residency or functional resource utilization. This symbolic-narrative mechanism integrates digital cultural consumption geographies (Wang et al., 2026) and the prioritization of urban identity in globalized contexts (Shaham-Maymon et al., 2025). Third, the serial path model of “Media Participation—Narrative Transportation—Place Identity—Alienation Association” provides an expandable framework for future research. By embedding this model within the concepts of time-geography (Dixon et al., 2022), future studies can conduct validation tests on this model in the context of different types of cultural engagement (such as local music, folk festivals) or different city sizes (Carrasco Cruz et al., 2025).

### 6.3 Practical implications and policy recommendations

The findings of this study have multi-level reference value for urban cultural policy and the construction of a psychological protection system for youth groups.

At the level of urban cultural brand building, the findings of this study support the policy value of the “literary origin protection and activation” strategy: a city’s literary heritage should not be regarded merely as material for tourism development but should be integrated into the daily cultural engagement scenes of its residents, whether they are newly arrived floating youth or long-term native residents. As the ANOVA findings in the empirical section of this study show, the sense of urban alienation has evolved into a universal psychological dilemma that transcends household registration and housing status. Therefore, when urban public cultural services play their psychological “stress-reducing and buffering” function, household registration status should no longer be the sole priority consideration. Existing practical cases such as the “Suzhou · Reader Program” and “Chengdu · City of Literature” all point in this direction.

Furthermore, policy initiatives for the construction of “Cities of Literature” should be systematically guided by a spatial equity perspective, focusing on the balanced distribution and physical accessibility of cultural resources. Rather than concentrating premium local literature IP assets and experiential cultural spaces solely in gentrified commercial districts, flagship downtown institutions, or historical tourist zones, municipal planners should decentralize these resources. Ensuring that community reading hubs, local narrative installations, and public literary salons are accessible in suburban areas, developmental sub-centers, and neighborhoods dominated by transient migrant populations is critical. By democratizing access to narrative place-making tools, local governments can ensure that cross-media literary development supports different urban districts and marginalized mobility groups rather than serving merely as a commodified marketing tool for elite consumption.

At the level of cross-media IP development, this study reminds practitioners that to effectively stimulate narrative transportation, it is necessary to go beyond superficial visual spectacles and focus on the in-depth restoration of the “localness” of literary works—the sound texture of a specific street, the phonetic texture of a specific dialect, the natural landscape of a specific season. These details are the psychological keys that trigger the audience to experience an “unfamiliar place” as a “hometown.” This is also crucial for tourism entrepreneurship that relies on place identity (Hallak, 2024).

At the level of youth mental health services, instead of relying solely on explicit counseling channels such as psychological counseling or community activities, incorporating the accessibility of local cultural resources into the construction indicators of a “mental health-friendly city” may be a more universally beneficial and sustainable path.

### 6.4 Research limitations and future outlook

Any cross-sectional survey research faces common methodological constraints, and this study is no exception.

First, the cross-sectional design precludes establishing a definitive temporal sequence between variables; all paths in this study are described in terms of statistical prediction and correlation rather than strict causality. Because place identity formation and the experiential alleviation of alienation are dynamic processes that unfold over time and across diverse contexts, this cross-sectional approach cannot capture time-varying patterns or spatial heterogeneity. Future research should adopt longitudinal tracking designs or mixed-methods approaches—combining quantitative spatial tracking with qualitative in-depth interviews—to capture these spatio-temporal dynamics.

Second, although Harman’s single-factor method was used to check for common method bias, due to the homogeneity of the data source (all self-reported), some measurement errors are still difficult to completely control. Future research could consider a multi-source fusion design that combines objective behavioral data (such as platform reading time logs, on-site check-in records) with subjective report data.

Third, the sampling of this study was conducted through the Wenjuanxing platform, and the respondents’ level of digital media use is generally high. This sampling bias limits the representativeness of the findings for groups with diverse spatio-temporal behavioral patterns, such as digitally marginalized populations or manual labor migrants, thus constraining the external validity of the model. For example, for youth groups who aspire to return to the countryside, the formation mechanism of their place attachment may be different (Carrasco-Cruz and Cruz-Souza, 2025).

Fourth, the five engagement dimensions of the CMLLE scale (in-depth text reading, visual narration, digital social interaction, immersive audio, embodied practice) were entered into the analysis in the form of a composite total score in this study, without a fine-grained comparison of the differential contributions of each dimension. Future research should propose and test specific hypotheses regarding the distinct spatio-temporal properties of these dimensions. For instance, future work could investigate whether virtual digital interactions primarily facilitate globalized social support networks, whereas physical embodied practices (such as literature-driven mobility trajectories) exert a stronger and more immediate impact on physical place identity.

## Data Availability

The original contributions presented in the study are included in the article/Supplementary material, further inquiries can be directed to the corresponding author.

## Footnotes

1 Blossoms Shanghai is a television series directed by Wong Kar-wai, officially released on December 27, 2023, and concluded in early 2024. Set in 1990s Shanghai, it depicts the city’s rapid economic rise and social transformation, centering on themes of commercial ambition, personal striving, and nostalgic urban memories.

2 My Altay is a television miniseries directed by Teng Congcong and released in May 2024. The story explores the themes of ethnic integration, coexistence with nature, and spiritual healing through the lived experiences of a young Han Chinese woman in the pastures of the Altay region, Xinjiang.
