# Supplementary material for: A study on the relationship among cross-media local literature engagement, place identity, and urban alienation in youth groups
Source: Front Psychol. 2026 Jul 20;17:1880209. doi: 10.3389/fpsyg.2026.1880209 (PMC13429646; doi:10.3389/fpsyg.2026.1880209)
Supplement: Supplementary file 1 [file Data_Sheet_1.ZIP › Supplementary_Material_1_Research_Instruments.docx]

# Supplementary Material 1: Research Instruments

## Part I: Participant Information and Informed Consent

**Dear Participant,**
You are invited to take part in an academic study exploring the psychological impact of local literature engagement on urban youth. This survey is conducted solely for academic research purposes.

**Anonymity & Privacy**: Your responses are completely anonymous. No personally identifiable information will be collected.

**Voluntary Participation**: Your participation is voluntary. You may withdraw at any time by closing the survey window.

**Estimated Time**: The survey takes approximately 3–5 minutes to complete.

**Attention Check**: This survey includes a quality control item. Please read each item carefully.

**Do you agree to participate?**
[ ] Yes, I consent.
[ ] No (Exit survey).

## Part II: Demographic Profile (DEMO)

**1.Gender**: [1] Male [2] Female [3] Non-binary/Prefer not to say

**2.Age**: (Numeric, range 18-40)

**3.Educational Attainment**: [1] Associate degree or below [2] Bachelor’s degree [3] Postgraduate or above

**4.Residential Identity**:

[1] Native Resident (Born and raised locally)

[2] Long-term Settler (Lived here > 3 years, non-local Hukou)

[3] Short-term Migrant (Lived here < 3 years)

[4] Digital Nomad (Remote worker with no fixed city)

**5.Housing Stability**: [1] Owned [2] Long-term Renting [3] Short-term/Shared Renting [4] Dormitory/Other

## Part III: Measurements (Likert 1-5 Scale)

*(*1 = *Never/Strongly Disagree,* 5 = *Very Frequently/Strongly Agree)*

### 1. Cross-media Local Literature Engagement (CMLLE)

*Instructions: Please rate the frequency of your engagement with local literature related to your current city in the past 12 months.*

**CMLLE1**: Actively read original novels, poems, or essays set in my current city.

**CMLLE2**: Watch films, TV series, or documentaries adapted from local literature.

**CMLLE3**: Participate in or search for discussions about local literary works on social media (e.g., Little Red Book, Douban).

**CMLLE4**: Listen to audiobooks, podcasts, or radio dramas related to local city stories.

**CMLLE5**: Physically visit streets, historic buildings, or specific shops mentioned in literary works.

**CMLLE6**: Purchase cultural creative products or merchandise related to local literary IPs.

**CMLLE7**: Plan Citywalk routes based on descriptions in literary works.

### 2. Narrative Transportation Experience (LNTS)

*Instructions: Please recall your psychological state during your most recent engagement with the works mentioned above.*

**LNTS1**: I felt as if I had “transported” into the world depicted in the story.

**LNTS2**: I could easily visualize the city streets and atmosphere described in the work.

**LNTS3**: My attention was fully absorbed; I was hardly distracted by the real world.

**LNTS4**: I felt strong emotional resonance with the fates of the characters in the story.

**LNTS5**: After finishing the work, the “cultural resonance” of the city lingered in my mind for a long time.

### 3. Literature-Based Place Identity (LBPIS)

*Instructions: Evaluate the impact of literary engagement on your relationship with this city.*

**LBPIS1**: Literary narratives make me feel that this city is a spiritual home rather than just a workplace.

**LBPIS2**: I feel that the city’s cultural temperament has become part of my self-identity.

**LBPIS3**: Learning the city’s cultural lineage through literature gives me a sense of rootedness.

**LBPIS4**: Compared to economic landmarks, those locations with literary heritage are the core of my city identity.

**LBPIS5 (Attention Check)**: To ensure data quality, please select “Strongly Agree” (5) for this item.

**LBPIS6 (Reverse)**: Without these cultural stories, this city would be no different from any other industrial city to me.

### 4. Multidimensional Urban Alienation (MDUAS)

*Instructions: Please evaluate your overall psychological experience in the current city.*

**MDUAS1**: I often feel a profound sense of isolation even in crowded commercial areas.

**MDUAS2**: It is difficult to find friends here with whom I can share deep emotional or cultural resonance.

**MDUAS3**: Faced with high living costs and competition, I feel powerless to control my own destiny.

**MDUAS4**: I often feel like a replaceable cog in a massive urban machine.

**MDUAS5**: The fast-paced, repetitive life makes me doubt the ultimate meaning of my efforts.

**MDUAS6**: Homogenized shopping malls and streets make the urban space feel cold and soulless.

**MDUAS7**: Rapid urban renewal causes me to feel a sense of “familiar strangeness” toward my neighborhood.

**MDUAS8**: I find it difficult to express my true self while trying to adapt to this city’s life.

**MDUAS9 (Reverse)**: I can find a personal time and space that makes me feel relaxed and fulfilled in the current urban rhythm.

# Data Coding Book (Codebook)

| Variable Category | Code | Item Description | Coding / Values | Recoding Instructions |
| --- | --- | --- | --- | --- |
| Demographics | GENDER | Gender | 1=Male, 2=Female, 3=Other | N/A |
|  | AGE | Age | Numeric value (18-40) | N/A |
|  | EDU | Education | 1=Associate/Below, 2=Bachelor, 3=Postgrad | N/A |
|  | IDENTITY | Residential ID | 1=Native, 2=Long-term, 3=Short-term, 4=Nomad | N/A |
|  | HOUSE | Housing | 1=Owned, 2=Long-rent, 3=Short-rent, 4=Dorm | N/A |
| Independent Var | CMLLE | Cross-media Engagement | Mean of CMLLE1 to CMLLE7 | Likert 1-5 |
| Mediator 1 | LNTS | Narrative Transportation | Mean of LNTS1 to LNTS5 | Likert 1-5 |
| Mediator 2 | LBPIS | Place Identity | Mean of LBPIS1, 2, 3, 4, 6 | LBPIS6: 6 - x (Reverse) |
| Dependent Var | MDUAS | Urban Alienation | Mean of MDUAS1 to MDUAS9 | MDUAS9: 6 - x (Reverse) |
| Quality Control | QC_ATT | Attention Check | Item LBPIS5 | Exclude if QC_ATT ≠ 5 |
